# Rezidivierende Larynxpapillomatose

**DOI:** 10.1007/s00106-022-01250-1

**Published:** 2022-12-07

**Authors:** Annekatrin Coordes, Daniel Grund, Alexander Mainka, Heidi Olze, Leif Hanitsch, Horst von Bernuth, Steffen Dommerich

**Affiliations:** 1 Klinik für Hals‑, Nasen- und Ohrenheilkunde, Campus Virchow-Klinikum and Campus Charité Mitte, Charité – Universitätsmedizin Berlin, Corporate Member der Freien Universität Berlin and Humboldt-Universität zu Berlin, Augustenburger Platz 1, 13353 Berlin, Deutschland; 2 Medizinische Klinik mit Schwerpunkt Infektiologie und Pneumologie der Charité, Charité – Universitätsmedizin Berlin, Corporate Member der Freien Universität Berlin and Humboldt-Universität zu Berlin, Augustenburger Platz 1, 13353 Berlin, Deutschland; 3 Klinik für Audiologie und Phoniatrie, Charité – Universitätsmedizin Berlin, Corporate Member der Freien Universität Berlin and Humboldt-Universität zu Berlin, Augustenburger Platz 1, 13353 Berlin, Deutschland; 4 Institut für Medizinische Immunologie an der Charité Berlin, Charité – Universitätsmedizin Berlin, Corporate Member der Freien Universität Berlin and Humboldt-Universität zu Berlin, Augustenburger Platz 1, 13353 Berlin, Deutschland; 5 Klinik für Pädiatrie mit Schwerpunkt Pneumologie, Immunologie und Intensivmedizin, Charité – Universitätsmedizin Berlin, Corporate Member der Freien Universität Berlin and Humboldt-Universität zu Berlin, Augustenburger Platz 1, 13353 Berlin, Deutschland

**Keywords:** Rezidivierende Larynxpapillomatose, HPV, Gardasil, Impfung, Bevacizumab, Cidofovir, Recurrent laryngeal papillomatosis, Human papillomavirus, Gardasil, Vaccination, Bevacizumab, Cidofovir

## Abstract

Die rezidivierende Larynxpapillomatose (RLP) wird in 90 % der Fälle durch die humanen Papillomviren (HPV) 6 und 11 verursacht. Unklar ist, ob Rezidive durch Neuinfektion oder Ausbreitung infizierter Zellen entstehen. Symptomatische und z. T. kurative Therapie ist die laserchirurgische bzw. konventionelle mikrochirurgische Abtragung. Die Operation zielt auf die Linderung der Atemnot und Verbesserung der Stimme. Im Krankheitsverlauf werden Patienten, insbesondere Kinder, durch Stimmprobleme, wiederholte operative Abtragungen, pulmonale Manifestationen und psychologische Traumata beeinträchtigt. Die Impfung mit Gardasil 9 (Merck & Co., Rahway, NJ, USA) beugt Neuinfektionen mit HPV 6, 11, 16, 18, 31, 33, 45, 52 und 58 vor und induziert Impfantigen-spezifische Antikörper und CD4+-T-Helferzellen. Die RLP ist nach aktueller Studienlage durch eine prophylaktische Impfung vermeidbar. Die Behandlung ist mit dem allgemeinen Impfrisiko verbunden (EMA-Zulassung: Mädchen, Jungen ab 9 Jahren). Studien zeigen zudem, dass der Impfstoff *nach* Entfernung HPV-assoziierter Neoplasien/Papillome Rezidiven vorbeugt. Die Erweiterung der Impfempfehlung für die Rezidivprophylaxe HPV-assoziierter Erkrankungen und als prophylaktische Impfung bei Männern würde zusätzlich die Anwendbarkeit und Herdenimmunität fördern. Für seltene und therapieresistente Fälle mit laryngotrachealer Beteiligung ist die systemische Therapie mit Bevacizumab (Avastatin; Genentech, San Francisco, CA, USA), einem VEGF-Antikörper, eine vielversprechende adjuvante Therapiemöglichkeit.

## Abstract (en)

Human papillomaviruses (HPV) 6 and 11 cause 90% of recurrent laryngeal papillomatosis (RLP). It is unclear whether recurrences are caused by new infections or the spread of infected cells. Symptomatic and sometimes curative treatment is laser surgery or conventional microsurgical removal. RLP surgery aims to relieve shortness of breath and improve the voice. Patients (especially children) are affected by voice problems, repetitive surgeries, pulmonary manifestations, and psychological trauma. Vaccination with Gardasil 9 (Merck & Co., Rahway, NJ, USA) prevents new infections with HPV 6, 11, 16, 18, 31, 33, 45, 52, and 58 and induces vaccine antigen-specific antibodies and CD4+ T helper cells. According to current studies, RLP can be avoided with prophylactic vaccination. The treatment is associated with a general vaccination risk (European Medicines Agency approval: girls and boys from 9 years). Studies also show that the vaccine *after *removal of HPV-associated neoplasia/papilloma prevents recurrences. Extension of the vaccination recommendation to prevent recurrences of HPV-associated diseases in men may promote applicability and herd immunity. For rare and treatment-refractory cases with laryngotracheal involvement, systemic therapy with bevacizumab (e.g. Avastin; Genentech, San Francisco, CA, USA), a VEGF antibody, is a promising adjuvant treatment option.

## Hintergrund

Die rezidivierende Larynxpapillomatose (RLP) tritt mit zwei Häufigkeitsgipfeln als juvenile und adulte Form auf. Sie wird in über 90 % durch humane Papillomviren (HPV) Typ 6 und 11, also Niedrigrisiko-HPV, hervorgerufen [1] und nur sehr selten durch die karzinogenen Typen HPV 16 und 18. Bei der juvenilen Form wird eine Infektion perinatal [2] bzw. in der Kindheit als Ursache angesehen, während bei der adulten Form eine Übertragung durch sexuelle Kontakte als wahrscheinlich gilt.

Bei aggressiven Verlaufsformen werden die Patienten mit wenigen Wochen Abstand z. T. mit Luftnot/Erstickungsgefahr in der Klinik vorstellig. Umgehend erfolgt dann eine Notfalloperation zur Sicherung der Atemwege. In seltenen Fällen kann eine Tracheotomie erforderlich sein, die nach Möglichkeiten verhindert werden sollte, da sich nachfolgend die Papillome häufig in der Trachea und der Lunge ausbreiten. Die wiederholten operativen Papillomabtragungen können zu bleibenden Stimm- und Atemstörungen durch Vernarbungen führen und so sekundär die Lebensqualität beeinträchtigen. Ebenso werden die Lebenserwartung und körperliche Entwicklung negativ beeinflusst. Weitere Beeinträchtigungen entstehen durch extralaryngeale Ausbreitungen im Bronchialsystem. Je früher die Erkrankung auftritt, umso aggressiver sind die Verläufe [3, 4].

**Figure 1:**
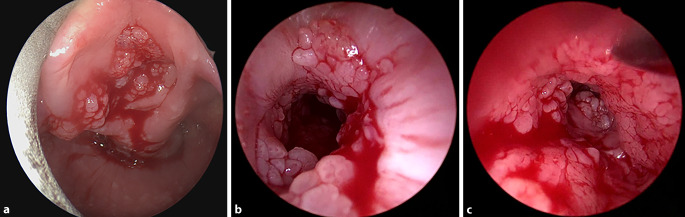

Der Verlauf der RLP ist nicht vorherzusehen. Eine Prognose kann man den Eltern bzw. Patienten nur schwer nennen. Insbesondere bei der juvenilen Form erreichen die Patienten sehr selten die Remission und leiden unter der Erkrankung bis ins Erwachsenenalter (Abb. 1). In diesem Zusammenhang ist ebenfalls unklar, ob Rezidive als Folge einer Neuinfektion von Nachbarzellen oder durch Ausbreitung bereits infizierter Zellen entstehen [5].

Um einem Notfalleingriff vorzubeugen kann man regelmäßige Operationen in Vollnarkose planen. Im Kindesalter können die Symptome als Asthma oder Krupp-Husten fehlgedeutet werden. Auch Erwachsene sind in ihrer Lebensführung, im Berufs- und im Familienleben beeinträchtigt.

Die RLP zählt zu den Präkanzerosen des Larynxkarzinoms. Bei langjährigen Verläufen können vor allem bei HPV-11-positiven Läsionen maligne Entartungen auftreten [6, 7]. Chirurgische Eingriffe können nicht der malignen Transformation vorbeugen [8]. Kommt es zur Mitbeteiligung des Lungenparenchyms, ist dies mit einer schlechten Prognose (z. B. Bronchialkarzinom) assoziiert [9–11].

Bei Patienten, die an einer HPV-assoziierten Erkrankung leiden, ist das Immunsystem nicht in der Lage, das Virus adäquat zu bekämpfen und die notwendigen Antikörper zu bilden. Patienten, die unter einem Immundefizit (z. B. HIV) leiden, erkranken häufiger an einer HPV-assoziierten Erkrankung als immunkompetente Patienten [12]. Bei der RLP ist ätiologisch bisher unklar, warum nur wenige Kinder eine RLP nach Exposition mit diesem relativ häufigen Virus entwickeln. Vermutlich weisen diese Kinder eine selektive Anfälligkeit für diese Erkrankung durch einen angeborenen Immundefekt auf (Inborn Error of Immunity). Ursächlich wurde bereits eine Mutation mit Gain of Function (GOF) in *NLRP1* beschrieben [13]. Eine homozygote NLRP1-Mutation führt zu einer erhöhten Inflammasom-Aktivität und wurde Geschwistern mit der klinischen Diagnose einer rezidivierenden Papillomatose ohne Nachweis einer HPV-Infektion identifiziert. Andere Untersuchungen konnten diese Mutation nicht identifizieren [14] und fanden dafür eine erhöhte Expression von IL‑8 und VEGF [14–17]. In Anbetracht der erhöhten VEGF-Expression ist wahrscheinlich, dass bestimmte Patienten mit RRP-Läsionen von einer Behandlung mit Bevacizumab profitieren.

Die aktuelle Standardbehandlung basiert auf der mikrolaryngoskopischen chirurgischen Abtragung, welche entweder konventionell mit abtragenden Instrumenten oder laserchirurgisch mit einem CO_2_-Laser erfolgen kann. Als Alternative kann eine ambulante Laser-Operation mit einem photoangiolytischen Laser (KTP-Laser oder Blue Laser) in oberflächlicher Sprühbetäubung erfolgen. Die operativen Therapien zielen derzeit auf eine Linderung der Atemnot und Verbesserung der Stimme in Sinne einer symptomatischen Therapie. Ob durch diese Therapie auch eine Heilung zu erwarten ist, kann nicht vorhergesagt werden.

## Prävention

Kinder mit RLP haben häufig keine Serumantikörper gegen die beteiligten HPV-Typen 6 und/oder 11 [10]. Ein signifikant höherer HPV-11-Antikörper-Titer wurde bei Kindern gemessen, die mindestens ein Jahr rezidivfrei waren im Vergleich zu Kindern mit aktiver Erkrankung [18]. Denkbar ist, dass neutralisierende Antikörper auf den Epitheloberflächen einen hemmenden Effekt auf die Proliferation, Ausbreitung oder Neubildung von HPV-Läsionen haben.

Auch die Impfung führt zur Bildung neutralisierender Antikörper. Daher ist nach aktueller Studienlage die Low-Risk-HPV-6/11-Infektion – als Ursache der RLP – durch eine prophylaktische Impfung vermeidbar. Seit 2006 ist der polyvalente Impfstoff Gardasil (Fa. Merck) verfügbar, der auf dem Hauptkapsidprotein L1 der vier HPV-Typen 6, 11, 16 und 18 basiert. Ein weiterer Impfstoff ist Cervarix (Fa. GlaxoSmithKline), der 2007 in Deutschland zugelassen wurde und auf den L1-Protein der Subtypen HPV 16 und 18 basiert. Der neunfache polyvalente Impfstoff (Gardasil 9) umfasst inzwischen zusätzlich auch die Subtypen HPV 31, 33, 45, 52 und 58. Durch die Impfung werden virusneutralisierende Antikörper gegen das Viruskapsid L1 stimuliert. Der Impfstoff induziert bei gesunden Probanden hohe Impfantigen-spezifische Antikörper sowie Impfantigen-spezifische CD4+-T-Helferzellen [19, 20] und verhindert Neuinfektionen [21].

In Deutschland wird die Impfung vom Robert Koch-Institut für Jungen und Mädchen zwischen 9 und 14 Jahren mit zwei Dosen im Abstand von mindestens 5 Monaten empfohlen. Bei kürzerem Abstand oder einem Alter ab 15 Jahren werden drei Dosen empfohlen (0-1-6 Monate für Cervarix und 0‑2-6 Monate für Gardasil). Die Impfquote liegt in Deutschland bei 31,3 % [22]. Im Vergleich dazu liegt die Impfquote in Australien bei 80 % [23].

In Australien konnte so bereits eine rückläufige Inzidenz der RLP belegt werden [24]. Demnach sank die Häufigkeit von 0,16 auf 0,02 pro 100.000 Kinder, sodass dort nur 15 neue Fälle innerhalb eines Jahres gemeldet wurden. Sinkende Inzidenzen wurden auch in Kanada und den USA beschrieben. Allerdings beträgt auch in den USA die Prävalenz der Immunisierung im Alter von 11 bis 17 Jahren (im Jahr 2018) lediglich 68 % (für eine Dosis); nur 51 % der Kinder haben die Impfung tatsächlich abgeschlossen [25]. Eine Fokussierung auf der HPV-Impfung als Anti-Krebs-Impfstoff in sozialen Medien könnte ein effektiver Weg für eine höhere Impfrate sein [26, 27].

In der Gynäkologie hat sich, basierend auf der bekannten Assoziation des Zervixkarzinoms mit HPV-Infektionen (insbesondere HPV 16 und 18), ein erfolgreicher Präventionsansatz zur Reduktion der Zervixkarzinominzidenz ergeben. Die prophylaktische Impfung beugt laut der aktuellen Studienlage einer Infektion mit HPV-Typen des Impfstoffs und Entwicklung von genitalen Dysplasien für mindestens 9,4 Jahre vor [28].

Im HNO-Bereich wird die zunehmende Inzidenz der Oropharynxkarzinome durch die Pathogenese mit HPV 16 erklärt [29]. Bei einem Auftreten im Alter von 55–65 Jahren wird der Effekt der Impfung sich erst in den zukünftigen Jahren zeigen und ist durch die geringe Impfquote in Deutschland auch nur abgeschwächt zu erwarten.

## Impfung als Therapie

Neben der Prävention kann die HPV-Impfung eine gewisse Rolle bei der Behandlung von RLP spielen. Die Boosterung mit dem HPV-Impfstoff soll bei Patienten mit RLP, die bereits mit HPV infiziert sind, zu einer Verbesserung der Immunität und der klinischen Symptomatik führen und somit auch eine therapeutische Wirkung bei dieser Erkrankung aufweisen [30, 31]. Die Induktion einer effektiven Immunität durch HPV-Impfung kann zu einer Eindämmung, Verlangsamung des Wiederauftretens oder möglicherweise zu einer Ausheilung führen. Naturgemäß wurden Impfungen bei jungen Patienten nur in weit fortgeschritten Stadien mit hochdramatischen klinischen Verläufen (für Kind, Eltern und Ärzte) durchgeführt. In den letzten Jahren wurden mehrere Fallserien veröffentlicht, bei denen der Impfstoff Gardasil ergänzend zur chirurgischen Papillomentfernung verwendet wurde und das Intervall zwischen den chirurgischen Abtragungen reduziert hat [31–35]. Auch bei hochgradigen intraepithelialen Neoplasien des Analkanals und der Zervix, die operativ versorgt wurden, konnte er Rezidiven vorbeugen [36–38]. Auf vorbestehende genitale Läsionen hatte der Impfstoff allerdings keinen Einfluss [39]. Eine Metaanalyse von Rosenfield et al. [40], basierend auf 12 Publikationen (63 Patienten mit RLP), zeigte eine signifikante Abnahme der Operationen nach HPV-Impfung. Das mittlere interoperative Intervall verlängerte sich von 7 Monaten auf 34 Monate nach der Impfung. Dies führt neben einer deutlich erhöhten Lebensqualität auch zur Senkung der Behandlungskosten.

In mehreren großen randomisierten Studien von erwachsenen Patienten mit chronischer HPV-Infektion und HPV-Nachweis im Sputum konnte eine Serokonversion nach HPV-Immunisierung nachgewiesen werden [41–43]. Aktuell wird eine prospektive Studie in den USA zum Thema Prävention HPV-bedingter Folgeerkrankungen durch die Immunisierung durchgeführt (NIH Clinical Trials, V501-031). Zukünftig kann es zu Veränderungen der Impfempfehlungen durch die Ständige Impfkommission in Deutschland kommen. Durch ein verändertes Impfregime, das auch die Erwachsenen einschließt, könnte eine Herdenimmunität aufgebaut werden.

Bei RLP-Patienten kann die HPV-Impfung trotz persistierender Infektion in 50 % zur Serokonversion führen. Der genaue Mechanismus für eine therapeutische Wirkung des HPV-Impfstoffs als Therapeutikum ist unbekannt. Natürliche Infektionen führen bei RLP-Patienten zu einer schwachen T‑Zell-Immunität. Erst durch die Impfung entsteht eine vollständige Immunantwort [19, 20].

E6- und E7-Proteine, die HPV-Onkoproteine, werden früh im Replikationszyklus in der HPV-infizierten Zelle produziert und spielen eine wesentliche Rolle bei der Transformation durch HPV [44, 45]. Sie wurden bei HPV-bedingten Oropharynxkarzinomen nachgewiesen [12, 46]. Diese beiden Onkoproteine E6 und E7 weisen eine hohe Expression bei HPV-bedingten Tumoren und assoziierten Erkrankungen auf, was zu einem interessanten Ziel als therapeutisches Target macht [47]. Bei Personen mit HPV-assoziierten Erkrankungen ist das Immunsystem möglicherweise nicht in der Lage, eine angemessene Abwehr gegen E6- und E7-Proteine aufzubauen. Nach der Impfung mit dem L1-Kapsidprotein zeigten RLP-Patienten auch verstärkte HPV-6-E6/E7-Antworten [48]. Die auf diese Weise induzierte E6/E7-spezifischen TH1-T-Zellen haben möglicherweise eine therapeutisch ähnliche Wirkung und können somit das Wachstum rezidivierender Papillome verhindern. Damit könnte pathophysiologisch bei RLP-Patienten eine Dysfunktion oder Dysregulation von CD4+-T-Zellen zugrunde liegen, die die Viruseliminierung und das immunologische Gedächtnis beeinträchtigen.

Neben dem derzeit erhältlichen Impfstoff Gardasil 9 gibt es bedeutende Durchbrüche bei HPV-DNA-Impfstoffen. Gardasil 9 zielt auf das L1-Protein des viralen Kapsids, um neutralisierende Antikörper zu erzeugen, während die DNA-Impfstoffe auf die Onkoproteine E6 und E7 abzielen [49]. Dadurch könnte eine robustere immunologische Antwort der T‑Zellen bei der Behandlung von HPV-bedingten Krankheiten erzeugt werden. Der neuartige Impfstoff INO-3016 hat in vitro und in vivo eine immunologische Reaktion gezeigt und die Häufigkeit operativer Eingriffe signifikant verringert [47, 50]. Derzeit laufen Studien für HPV-DNA-Impfstoffe (z. B. die Studie der Fa. INOVIO, INO-3107, NCT04398433).

Langfristig könnten immunologische Erkenntnisse bei ausbleibender klinischer Besserung über die Häufigkeit von „Impfversagern“ gewonnen werden. Diese Impfversager könnten möglicherweise genetische Ursachen haben und durch eine HLA-Typisierung erfasst werden. Für folgende Immunisierungen könnte das bereits prognostische Relevanz haben. Bekannt ist dies bereits für die Assoziation mit der NLRP1-GOF-Mutation, die zu einem autosomal-rezessiv vererbten Syndrom führt [13].

## Fortschritt in der medikamentösen Therapie

Bei geringem Therapieansprechen auf die chirurgische Abtragung der Papillome wurden in der Vergangenheit unterschiedliche Strategien zur Reduktion der Rezidivrate verfolgt: Immunstimulation mit Interferon‑α [7] und photodynamische Therapien [51] zeigten keinen signifikanten Erfolg. Die lokale Applikation des antiviralen Nukleosidanalogons Cidofovir [52, 53] ist bis heute umstritten. Einerseits wurde wiederholt über gute Behandlungserfolge berichtet, nicht nur in der HNO-Heilkunde, sondern auch in der Gynäkologie. Andererseits besteht keine Zulassung, und Cidofovir kann potenziell erhebliche Risiken hervorrufen, insbesondere hinsichtlich der Nierenfunktion und der potenziellen Mutagenität der Substanz. In besonderen Situationen kann im Einzelfall entschieden werden.

In der kürzeren Vergangenheit wurde die adjuvante medizinische Behandlung für Patienten mit schwerer RLP deutlich ausgeweitet, auch wenn Bevacizumab keine Zulassung für die Indikation Kehlkopfpapillomatose hat. Die aus der Onkologie bekannte gezielte Biologika-Therapie mit monoklonalen Antikörpern beeinflusst pathologische Signalwege durch Immunmodulation, Checkpointhemmung und VEGF-Hemmung in Ergänzungen zur chirurgischen Behandlung. Die Therapie basiert auf der Beobachtung, dass VEGF in Papillomen erhöht exprimiert wird [14–17]. Bevacizumab (Avastin, Genentech) nutzt den Signalweg des vaskulären endothelialen Wachstumsfaktors (VEGF). Eine starke Expression von VEGF bei RLP-Patienten besteht im Papillomepithel und den darunterliegenden Endothelzellen. Bevastatin ist ein monoklonaler Antikörper, der an VEGF bindet und die Wechselwirkung mit seinem Rezeptor hemmt. Mehrere Studien haben die intraläsionale Injektion ausgewertet. Die Injektion führte zu einem verlängerten Intervall zwischen chirurgischen Behandlungen [54, 55], verminderter Krankheitsschwere und einer Verbesserungen der stimmbezogenen Lebensqualität [55]. Als Komplikation wurde ein Granuloma pyogenicum beobachtet [56].

Die intravenöse Gabe von Bevacizumab hat sich als eine vielversprechende adjuvante medizinische Behandlung für Patienten mit fortgeschrittener trachealer und pulmonaler RLP gezeigt (mindestens > 2 Eingriffe pro Jahr). In diesen Fällen führte die Behandlung zu einer signifikanten Regression bis zu einem vollständigen Verschwinden der Läsionen in Trachea und Larynx. Die Patienten wurden mit einer Dosierung von 5–10 mg/kg in 1,5 h alle 2 bis 4 Wochen behandelt. Die Intervalle wurden konsekutiv auf 2 bis 3 Monate verlängert und mit endoskopischen Kontrollen zur Bewertung der Läsionen verbunden. Regelmäßige Laborkontrollen sollten Nierenfunktion und Elektrolyte beinhalten sowie ein Echokardiogramm und Blutdruckkontrollen [57–60].

Eine internationale Konsenserklärung zur systemischem Therapie mit Bevacizumab von Sidell et al. [61] bietet derzeit eine vorläufige Leitlinien für diese Off-Label-Anwendung. Der Arzt muss in jedem Fall den Off-Label-Use mit dem Patienten besprechen und dokumentieren. Als weiterhin wichtig hervorzuheben ist, dass die intravenöse Bevastatin-Erhaltungstherapie keinen klaren Endpunkt hat.

Eine weitere zielgerichtete Therapie ist der monoklonale Antikörper, der auf den Signalweg 1 des programmierten Zelltods (PD-1) abzielt [62]. Dieser Signalweg hat klinische Aktivität bei HPV-assoziierten Kopf-Hals-Karzinomen gezeigt. PD-L1-Antikörper werden hier erfolgreich zur Behandlung eingesetzt. In einer Phase-II-Studie im Jahr 2019 wurden 12 erwachsene RLP-Patienten mit dem monoklonalen Anti-PD-L1-Antikörper Avelumab (z. B. Bavencio) behandelt. Alle RLP-Patienten mit Beteiligung des Larynx zeigten ein partielles Ansprechen, während Patienten mit pulmonaler Beteiligung keine Reaktion auf ihre Lungenläsionen aufwiesen [63].

## Fazit für die Praxis

- Die RLP wird in 90 % durch HPV 6 und 11 verursacht.
- Die Impfung mit Gardasil 9 beugt Neuinfektionen mit HPV 6, 11, 16, 18, 31, 33, 45, 52 und 58 vor (STIKO-Empfehlung).
- Die Impfung mit Gardasil 9 nach Entfernung HPV-assoziierter Neoplasien/Papillome kann Rezidiven vorbeugen (bisher Off-Label-Use).
- Für seltene/therapieresistente Fälle mit laryngotrachealer Beteiligung bietet Bevacizumab eine Möglichkeit für eine medikamentöse adjuvante Therapie.
- PD-L1-Antikörper kommen als Alternative vorläufig nur im Rahmen von Studien in Betracht.

## References

[1] Terry RM (1987). Demonstration of human papillomavirus types 6 and 11 in juvenile laryngeal papillomatosis by in-situ DNA hybridization. J Pathol.

[2] Alberico S (1996). Maternal-fetal transmission of human papillomavirus. Minerva Ginecol.

[3] Ruparelia S (2003). Predictors of remission in juvenile-onset recurrent respiratory papillomatosis. Arch Otolaryngol Head Neck Surg.

[4] Reeves WC (2003). National registry for juvenile-onset recurrent respiratory papillomatosis. Arch Otolaryngol Head Neck Surg.

[5] Pawlita M, Gissmann L (2009). Recurrent respiratory papillomatosis: indication for HPV vaccination?. Dtsch Med Wochenschr.

[6] Kimberlin DW (2004). Current status of antiviral therapy for juvenile-onset recurrent respiratory papillomatosis. Antiviral Res.

[7] Gerein V (2005). Incidence, age at onset, and potential reasons of malignant transformation in recurrent respiratory papillomatosis patients: 20 years experience. Otolaryngol Head Neck Surg.

[8] Stamataki S (2007). Juvenile recurrent respiratory papillomatosis: still a mystery disease with difficult management. Head Neck.

[9] Andratschke M, Betz C, Leunig A (2008). Laryngeal papillomatosis: etiology, diagnostics and therapy. HNO.

[10] Maloney EM (2006). Longitudinal measures of human papillomavirus 6 and 11 viral loads and antibody response in children with recurrent respiratory papillomatosis. Arch Otolaryngol Head Neck Surg.

[11] Boltezar IH (2011). Adjuvant therapy for laryngeal papillomatosis. Acta Dermatovenerol Alp Pannonica Adriat.

[12] Albers AE (2010). Prophylactic and therapeutic vaccines against human papilloma virus. HNO.

[13] Drutman SB (2019). Homozygous NLRP1 gain-of-function mutation in siblings with a syndromic form of recurrent respiratory papillomatosis. Proc Natl Acad Sci U S A.

[14] Sievers C (2021). Comprehensive multiomic characterization of human papillomavirus-driven recurrent respiratory papillomatosis reveals distinct molecular subtypes. Commun Biol.

[15] Rodman R (2014). Genetic dysregulation in recurrent respiratory papillomatosis. Laryngoscope.

[16] Rahbar R (2005). Role of vascular endothelial growth factor—a in recurrent respiratory papillomatosis. Ann Otol Rhinol Laryngol.

[17] DeVoti JA (2008). Immune dysregulation and tumor-associated gene changes in recurrent respiratory papillomatosis: a paired microarray analysis. Mol Med.

[18] Chen BB (2003). The detection and significance of human papilloma virus 11b virus like particles and its serum antibody in juvenile larynx papilloma. Zhonghua Er Bi Yan Hou Ke Za Zhi.

[19] Pacher SK (2011). Direkter longitudinaler Vergleich von T-Helfer-Zell Antworten auf prophylaktische HPV-Impfstoffe über einen Zeitraum von 12 Monaten.

[20] Ramseger A (2009). Messung zellulärer Immunantworten im Rahmen der prophylaktischen Impfung gegen Humane Papillomviren.

[21] Villa LL (2006). Immunologic responses following administration of a vaccine targeting human papillomavirus types 6, 11, 16, and 18. Vaccine.

[22] Robert Koch-Institut (2018) Aktuelles aus der KV-Impfsurveillance – Impfquoten ausgewählter Schutzimpfungen in Deutschland. Epidemiol Bull 01:8–9. https://www.rki.de/DE/Content/Infekt/EpidBull/Archiv/2018/Ausgaben/01_18.pdf?__blob=publicationFile

[23] Novakovic D (2018). A prospective study of the incidence of juvenile-onset recurrent respiratory papillomatosis after implementation of a national HPV vaccination program. J Infect Dis.

[24] Nunez CA (2018). Australian paediatric surveillance unit annual report 2018. Commun Dis Intell.

[25] Walker TY (2019). National, regional, state, and selected local area vaccination coverage among adolescents aged 13–17 years—United States, 2018. Mmwr Morb Mortal Wkly Rep.

[26] Massey PM (2020). Dimensions of misinformation about the HPV vaccine on Instagram: content and network analysis of social media characteristics. J Med Internet Res.

[27] Chen T, Dredze M (2018). Vaccine images on twitter: analysis of what images are shared. J Med Internet Res.

[28] Villa LL (2006). High sustained efficacy of a prophylactic quadrivalent human papillomavirus types 6/11/16/18 L1 virus-like particle vaccine through 5 years of follow-up. Br J Cancer.

[29] Mehanna H (2010). Oropharyngeal carcinoma related to human papillomavirus. BMJ.

[30] Mudry P (2011). Recurrent laryngeal papillomatosis: successful treatment with human papillomavirus vaccination. Arch Dis Child.

[31] Forster G (2008). Juvenile laryngeal papillomatosis—immunisation with the polyvalent vaccine gardasil. Laryngorhinootologie.

[32] Baumanis MM, Elmaraghy CA (2016). Intersurgical interval increased with use of quadrivalent human papillomavirus vaccine (Gardasil) in a pediatric patient with recurrent respiratory papillomatosis: a case report. Int J Pediatr Otorhinolaryngol.

[33] Fancello V (2015). HPV type 6 and 18 coinfection in a case of adult-onset laryngeal papillomatosis: immunization with Gardasil. Case Rep Otolaryngol.

[34] Meszner Z (2015). Recurrent laryngeal papillomatosis with oesophageal involvement in a 2 year old boy: successful treatment with the quadrivalent human papillomatosis vaccine. Int J Pediatr Otorhinolaryngol.

[35] Sullivan C, Curtis S, Mouzakes J (2017). Therapeutic use of the HPV vaccine in recurrent respiratory papillomatosis: a case report. Int J Pediatr Otorhinolaryngol.

[36] Joura EA (2007). Efficacy of a quadrivalent prophylactic human papillomavirus (types 6, 11, 16, and 18) L1 virus-like-particle vaccine against high-grade vulval and vaginal lesions: a combined analysis of three randomised clinical trials. Lancet.

[37] Kang WD, Choi HS, Kim SM (2013). Is vaccination with quadrivalent HPV vaccine after loop electrosurgical excision procedure effective in preventing recurrence in patients with high-grade cervical intraepithelial neoplasia (CIN2-3)?. Gynecol Oncol.

[38] Swedish KA, Factor SH, Goldstone SE (2012). Prevention of recurrent high-grade anal neoplasia with quadrivalent human papillomavirus vaccination of men who have sex with men: a nonconcurrent cohort study. Clin Infect Dis.

[39] Garland SM (2007). Quadrivalent vaccine against human papillomavirus to prevent anogenital diseases. N Engl J Med.

[40] Rosenberg T (2019). Therapeutic use of the human papillomavirus vaccine on recurrent respiratory papillomatosis: a systematic review and meta-analysis. J Infect Dis.

[41] Chaturvedi AK (2019). Prevalence of oral HPV infection in unvaccinated men and women in the United States, 2009–2016. JAMA.

[42] Handisurya A (2016). Human papillomavirus vaccination induces neutralising antibodies in oral mucosal fluids. Br J Cancer.

[43] Kahn JA (2015). Behavioral, immunologic, and virologic correlates of oral human papillomavirus infection in HIV-infected youth. Sex Transm Dis.

[44] Impfprävention HPV-assoziierter Neoplasien. S3 Leitlinie der Paul-Ehrlich-Gesellschaft für Chemotherapie (AG HPV-Management-Forum). http://www.awmf.org/uploads/tx_szleitlinien/082-002_S3_Impfpraevention_HPV-assoziierter_Neoplasien_06-2008_06-2013.pdf. Zugegriffen: 06.12.2022

[45] Stern PL (2012). Therapy of human papillomavirus-related disease. Vaccine.

[46] D’Souza G (2007). Case-control study of human papillomavirus and oropharyngeal cancer. N Engl J Med.

[47] Higgins LM (2016). Adolescents’ intention and self-efficacy to follow Pap testing recommendations after receiving the HPV vaccine. Hum Vaccin Immunother.

[48] Kaufmann AM (2022). Persönliche Mitteilung.

[49] Aggarwal C (2020). Immune therapy targeting E6/E7 oncogenes of human paillomavirus type 6 (HPV-6) reduces or eliminates the need for surgical intervention in the treatment of HPV-6 associated recurrent respiratory papillomatosis. Vaccines (Basel).

[50] Morrow MP (2016). Augmentation of cellular and humoral immune responses to HPV16 and HPV18 E6 and E7 antigens by VGX-3100. Mol Ther Oncolytics.

[51] Shikowitz MJ (2005). Clinical trial of photodynamic therapy with meso-tetra (hydroxyphenyl) chlorin for respiratory papillomatosis. Arch Otolaryngol Head Neck Surg.

[52] Wierzbicka M (2011). Effectiveness of cidofovir intralesional treatment in recurrent respiratory papillomatosis. Eur Arch Otorhinolaryngol.

[53] Cavel O (2012). Minimizing surgical management through the use of adjuvant medical therapies. Laryngoscope.

[54] Zeitels SM (2011). Local injection of bevacizumab (avastin) and angiolytic KTP laser treatment of recurrent respiratory papillomatosis of the vocal folds: a prospective study. Ann Otol Rhinol Laryngol.

[55] Maturo S, Hartnick CJ (2010). Use of 532-nm pulsed potassium titanyl phosphate laser and adjuvant intralesional bevacizumab for aggressive respiratory papillomatosis in children: initial experience. Arch Otolaryngol Head Neck Surg.

[56] Zelenik K (2021). Local bevacizumab treatment of juvenile-onset respiratory papillomatosis might induce multiple tracheal pyogenic granulomas. Laryngoscope.

[57] Best SR, Mohr M, Zur KB (2017). Systemic bevacizumab for recurrent respiratory papillomatosis: a national survey. Laryngoscope.

[58] Zur KB, Fox E (2017). Bevacizumab chemotherapy for management of pulmonary and laryngotracheal papillomatosis in a child. Laryngoscope.

[59] Ryan MA (2021). Systemic bevacizumab (avastin) for juvenile-onset recurrent respiratory papillomatosis: a systematic review. Laryngoscope.

[60] Tkaczuk A (2021). Parenteral bevacizumab for the treatment of severe respiratory papillomatosis in an adult population. Laryngoscope.

[61] Sidell DR (2021). Systemic bevacizumab for treatment of respiratory papillomatosis: international consensus statement. Laryngoscope.

[62] Ferris RL (2016). Nivolumab for recurrent squamous-cell carcinoma of the head and neck. N Engl J Med.

[63] Allen CT (2019). Safety and clinical activity of PD-L1 blockade in patients with aggressive recurrent respiratory papillomatosis. J Immunother Cancer.

